# Editorial: New insights into molecular mechanisms and targeted therapy for gastrointestinal tumors

**DOI:** 10.3389/fcell.2023.1289530

**Published:** 2023-09-28

**Authors:** Haoran Feng, Haosheng Li, Mengdi Chen, Tao Zhang, YiMing Zhou, Ye Xu, Wei Zhang, Tao Zhang

**Affiliations:** ^1^ Department of General Surgery, Ruijin Hospital, Shanghai Jiao Tong University School of Medicine, Shanghai, China; ^2^ Shanghai Institute of Digestive Surgery, Ruijin Hospital, Shanghai Jiao Tong University School of Medicine, Shanghai, China; ^3^ Human Oncology and Pathogenesis Program, Memorial Sloan Kettering Cancer Center, New York, NY, United States; ^4^ Department of General Surgery, Huashan Hospital, Fudan University, Shanghai, China; ^5^ Department of Colorectal Surgery, Fudan University Shanghai Cancer Center, Shanghai, China; ^6^ Department of Colorectal Surgery, Changhai Hospital, Naval Medical University, Shanghai, China

**Keywords:** gastrointestinal tumor, tumor microenvironment, antitumor therapeutic targets, mechanisms of tumorigenesis, novel therapeutic approach

Gastrointestinal (GI) malignancies account for nearly one-third of all cancer-related fatalities worldwide, including esophageal, gastric, colorectal, pancreatic, ampullary, biliary, and small intestinal carcinomas (Siegel et al., 2022). It is estimated that by the year 2040, the global burden of GI malignancies will escalate to 7.5 million new cases and 5.6 million fatalities (Arnold et al., 2020). Traditionally, the therapeutic approach for these tumors has been dominated by surgery, chemotherapy, and radiation therapy (Schrag et al., 2023). However, these treatments often pose challenges like systemic toxicity, suboptimal sensitivity, and the emergence of drug resistance (Vodenkova et al., 2020). The prelude of the “modern” era for the treatment of GI tumor treatment is characterized by the elucidation of molecular mechanisms underlying tumor development and the exploitation of targeted therapies (Sumiyoshi et al., 2023). Crucially, the indications for targeted drug interventions have shifted from anatomical origin of the tumor towards molecular diagnostics (Jung et al., 2020). Comprehensive molecular analysis has become indispensable for patient management across all solid GI tumors. Current challenges in treatment include the identification of novel therapeutic targets, the application of new pharmacological agents, and the initiation of exploratory clinical trials (El-Deiry et al., 2019). In-depth investigations into genomic, transcriptomic, and proteomic molecular mechanisms that drive tumor growth and progression are facilitating drug development (Sveen et al., 2020), while the deployment of monoclonal antibodies and small molecule inhibitors through clinical trials is improving overall survival (OS) for a broader patient population (Guan et al., 2023). This Journal Research Topic includes eight illuminating articles on the molecular mechanisms and targeted treatments for GI tumors.

At the molecular level, unveiling gene landscapes offers promising avenues for targeted therapies in patients with somatic mutations in key genes such as BRCA, KRAS, HER2, and mismatch repair proteins (Redegalli et al., 2022). Kumar et al. reported that in a cohort of 9,444 advanced Pancreatic Ductal Adenocarcinoma (PDAC) patients, those with wild-type KRAS exhibited a higher frequency of genomic alterations, providing more options for targeted treatments. Patients with lower TP53 mutation rates and higher tumor mutational burdens were found to be more responsive to immune checkpoint inhibitors (ICIs). Targeted therapies could improve survival outcomes in KRAS wild-type PDAC patients. Chemo-resistance has been a long-standing challenge in the treatment of various cancers, including pancreatic cancer (Daylami et al., 2014). To address this Research Topic, Chen et al. conducted transcriptomic analyses on samples from TCGA and GEO databases, identifying prognostic gene signatures associated with chemoresistance in pancreatic cancer. They constructed a predictive model hinging on five gene markers: EGFR, MSLN, ERAP2, ALDH3B1, and NCEH1, revealing their role in predicting chemoresistance, tumor mutational load, and immune cell infiltration. Single-cell RNA sequencing analyses revealed high expression of all five genes in tumor samples. *In vitro* experiments indicated that ALDH3B1 and NCEH1 were involved in pancreatic cancer progression and gemcitabine resistance. The identification of these potential therapeutic targets could pave the way to overcome chemotherapy resistance and improve overall prognosis in pancreatic cancer patients. The role of non-coding RNAs in regulating various biological processes within tumors is gaining attention (Soureas et al., 2023). Zhao et al. focused on the RNA-induced silencing complex as a diagnostic marker and therapeutic target in colorectal cancer (CRC). Utilizing bioinformatic and Cox proportional hazards regression analyses, a prognostic model was developed based on differentially expressed mRNAs and miRNAs between CRC and normal tissues. Moreover, POU4F1, DNASE1L2, and WDR72 were identified as potential miRNA target genes through immunohistochemical staining of tissue microarrays, offering novel avenues for RNA-based therapies in CRC. Understanding these interactions enables researchers to develop RNA-based therapeutic targets, providing new treatment strategies for patients with CRC.

At the level of known protein functions and signaling pathways, phosphorylated FAK (p-FAK) is closely associated with various malignancies (Oudart et al., 2017). The role of p-FAK in CRC remains controversial. Yu et al. elucidated the prognostic significance of p-FAK in CRC by conducting a comprehensive study involving 908 patients. Using immunohistochemistry, Kaplan-Meier survival analysis, and Cox regression models, a correlation was established between p-FAK expression and the clinical features of CRC patients. They found that high p-FAK expression level served as an independent risk factor and prognostic biomarker for CRC, predicting poor responsiveness to chemotherapy. This study underscores the potential of p-FAK as a novel therapeutic target in CRC. Simultaneously, molecular therapies have also been clinically validated for the treatment of gastric cancer (GC). However, the heterogeneity in GC subtypes and clinical manifestations poses certain challenges for treatment and prognosis (Lim et al., 2016). Dong et al. identified that MYL9 could promote the migration and metastasis of GC cells. Based on the multiple bioinformatic databases and the last absolute shrinkage and selection operator Cox regression, a prognostic model was constructed for GC patients. MYL9, the highest weight gene in the prognostic score, was correlated with worse clinical outcomes. This study provides a novel strategy for the development of treatments for metastatic GC patients. It has been discovered that the secretion of chemokines is crucial in the recruitment of immune cells and significantly impacts the progression of various malignancies (Liu et al., 2023). Roberto et al. unveiled the immunomodulatory mechanisms of the CXCL12-CXCR4-CXCR7 axis and its potential interactions with the tumor microenvironment in PDAC. Multiple combinatorial therapeutic approaches were developed to overcome the “cold” immune environment of PDAC, including using CXCL12 axis inhibitors combined with anti-PD-1/PDL1 agents. Their findings emphasize the importance of understanding the tumor immune microenvironment for the development of treatments and offer potential avenues for targeted therapies.

Given the heterogeneity of cancer, the latest therapeutic approaches have the potential to bring the next breakthrough in cancer treatment (Ma et al., 2019). In an exploration of therapeutic drugs, Fan et al. reviewed the potential of Curcumin (CUR) as a therapeutic agent for GI cancers. CUR was a hydrophobic polyphenol extracted from the rhizomes of turmeric, exhibiting robust anti-cancer properties with minimal toxicity. CUR exerted anti-cancer effects with multifaceted antitumor mechanisms, ranging from the induction of apoptosis and autophagy, cell cycle arrest, inhibition of epithelial-mesenchymal transition, suppression of cellular invasion and migration, modulation of multiple signaling pathways, sensitization to chemotherapy, reversal of drug resistance, to the regulation of the tumor microenvironment. In addition to the exploration of new drugs, clinical therapies have also been breakthroughs. Nie et al. aimed to assess the clinical efficacy and safety of various second-line treatment strategies for patients with advanced or metastatic GC. In their observational multicenter real-world study, patients who had failed prior therapies were enrolled and treated with either chemotherapy, anti-angiogenic tyrosine kinase inhibitors (TKIs) plus chemotherapy, or TKIs plus ICIs. The study compared progression-free survival (PFS), objective response rate (ORR), disease control rate (DCR), OS, and drug toxicities across the three treatment groups. The results showed that anti-angiogenic TKIs combined with chemotherapy demonstrated superior second-line or subsequent therapeutic efficacy for advanced or metastatic GC patients with well-tolerated toxicity. However, the combination of TKIs and ICIs failed to show a clinical advantage over chemotherapy alone. This research provides valuable insights into the efficacy of combining TKIs with chemotherapy. Such personalized approaches have the potential to optimize treatment outcomes, ensuring that patients receive the most effective therapies.

In conclusion, the articles included in this Research Topic provide a new direction for the development of new targets for the treatment of GI tumors. At the same time, we believe that with the unremitting efforts of researchers all over the world, new targets as effective as HER2, EGFR, and PD-L1 will be identified, ushering in renewed hope for GI tumor patients globally. The relentless exploration of cancer’s molecular and genetic landscapes ensures a future where cancer can be effectively managed.
